# Liver angiosarcoma, a rare liver malignancy, presented with intraabdominal bleeding due to rupture- a case report

**DOI:** 10.1186/1477-7819-10-23

**Published:** 2012-01-26

**Authors:** Chin-Ying Chien, Cheng-cheng Hwang, Chun-nan Yeh, Huang-yang Chen, Jui-Teng Wu, Chan-Siu Cheung, Chih-Lang Lin, Cho-li Yen, Wen-yen Wang, Kun-Chun Chiang

**Affiliations:** 1 General surgery department, Chang Gung Memorial Hospital, 222, Mai-Chin Road, Keelung, Taiwan; 2 Department of Pathology, Chang Gung Memorial Hospital, 222, Mai-Chin Road, Keelung, Taiwan; 3 General surgery department, Chang Gung Memorial Hospital, 5, Fu-Hsing Street, Kwei-Shan, Taoyuan, Taiwan; 4 Department of Radiology, Chang Gung Memorial Hospital, 222, Mai-Chin Road, Keelung, Taiwan; 5 Department of Gastroenterolgy, Chang Gung Memorial Hospital, 222, Mai-Chin Road, Keelung, Taiwan; 6 Graduate Institute of Clinical Medical Sciences, College of Medicine, Chang Gung University,259 Wen-Hwa 1st Road, Kwei-Shan Tao-Yuan, Taiwan; 7 Department of Nursing, Chang Gung Memorial Hospital, 222, Mai-Chin Road, Keelung, Taiwan

**Keywords:** angiosarcoma, liver angiosarcoma, hepatectomy, embolization

## Abstract

Liver angiosarcoma is a rare disease, however it still ranks as the third of most common primary liver maligancies. The prognosis of liver angiosarcoma is very poor with almost all patients with this kind of disease die within 2 years after diagnosis. No specific symptoms and signs are closely associated with this disease. Here, we report a case presenting shock status at first due to rupture of liver angiosarcoma- induced internal bleeding. After emergent transarterial embolization (TAE), she received partial hepatectomy two weeks later. 4 months after operation, she is still with a good performance status without obvious recurrence or metastasis identified.

## Background

Hepatic angiosarcoma is a very rare disease, accounting for only 2% of primary liver malignancy [1-3]; however, it still ranks as the third place in the list of most common primary liver malignancies [2,3]. Hepatic angiosarcoma originates from endothelial cells and usually presents as an abdominal mass with unspecific symptoms and signs [4], making it difficult to diagnose in the early stage. The survival of hepatic angiosarcoma is very poor, which is attributable to its rapid progress, high recurrence rate, and resistant to traditional chemotherapy and radiotherapy [5-7]. Even liver transplantation could not benefit patients with liver angiosarcoma [8]. To date, the therapeutic guideline for liver angiosarcoma has not been set up; partial liver resection to remove tumor radically still remains to be the cornerstone of treatment options. Here we reported a case with primary liver angiosarcoma presenting with hemoperitoneum due to tumor rupture and active bleeding treated by TAE and subsequent partial liver resection.

## Case presentation

**Figure 1:**
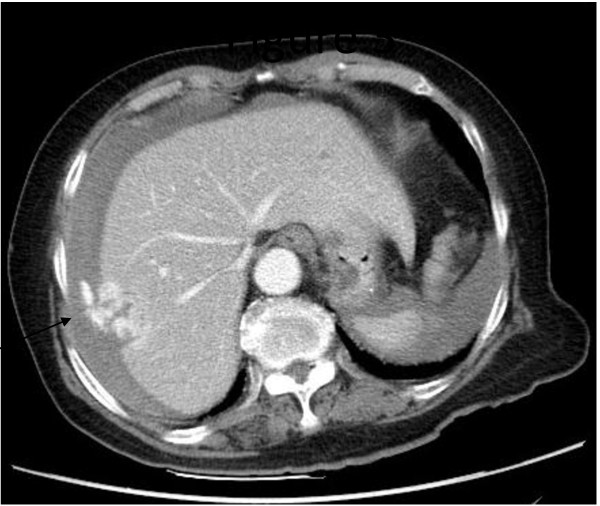
**Enhanced CT scan showed a ruptured irregular liver mass**. Enhanced CT scan showed a ruptured irregular liver mass (balck arrow) with prominent enhancement at the liver surface inducing contrast leakage into the right subphrenic space.

**Figure 2:**
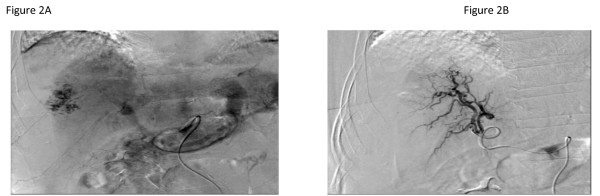
**The celiac trunk angiogram showed a hypervascular mass in right lobe of liver with contrast extravastion from the tumor surface (black arrow), which was compatible with CT findings of a hypervascular hepatic tumor bleeding. Figure 2B-After embolization, no contrast extravastion from the tumor was shown**.

An 83 year-old female complained about lower abdomen pain for two months. After one episode of fierce right upper abdomen pain occurred on July 12^th^, 2011, she was brought to our emergency department for aid by her families. In our emergency department (ER), unstable systolic blood pressure of 90 mmHg with tachycardia of 110 beats/min was found. Resuscitation was then given. Subsequent blood examination showed leukocytosis (161000/μL), anemia (9 g/dL), and thrombocytopenia (85000/μL). Abdomen distension with mild tenderness while pressed was also noted. Under the impression of internal bleeding, bedside echo was then conducted, which revealed intra-abdomen fluid accumulation and a liver mass. Abdomen computer tomography(CT) was thereafter arranged for this patient with contrast injection, which indicated a 3 cm hypervascular mass over segment 5 of liver with active bleeding (Figure 1). An emergent angiogram of celiac trunk was then conducted and showed a hypervascular tumor at the right lobe of liver which was supplied by the right hepatic artery. Extravastion of contrast medium from the tumor surface was shown which indicated an active tumor bleeding (Figure 2A). Then emergent embolization was done after superselective cannulation into the feeders using a 3 Fr. Microcatheter from the S5 branch of the right hepatic artery, 5 ml suspension of gelfoam powder (200 um) mixed with contrast medium were infused via the feeders into the right lobe tumor until the flow stasis. Post-embolization angiogram showed that no extravastion of the contrast medium from the right lobe tumor (Figure 2B). After admission, thorough liver function tests and hepatitis survey were done. This patient's liver function was categorized as Child Pugh A and no hepatitis was diagnosed. Alpha-fetoprotein, CEA, CA 19-9 were within normal range. Under the impression of liver tumor, highly suspected as a hepatoma based on findings in TAE and abdominal CT of this patient, laparotomy were performed two weeks later after TAE. During operation, 2000 cc bloods inside abdomen was found. A 3 cm tumor over surface of segment 5 of liver was noted. Partial hepatectomy of liver segment 5 to remove tumor was done.

### Pathological examination

**Figure 3:**
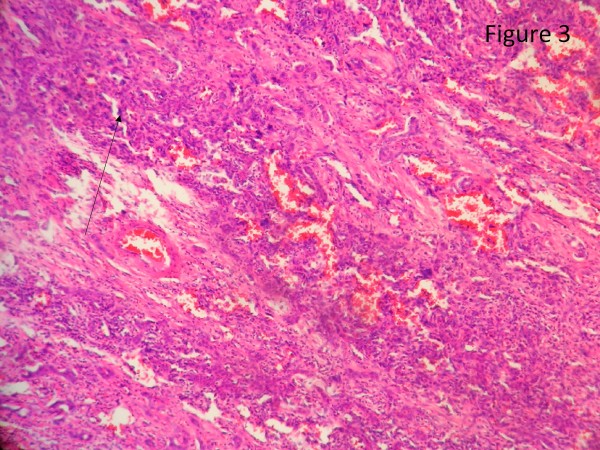
**The section of resected liver showed a tumor composed of anastomosing vascular channels in an infiltrating growth pattern**. The section of resected liver showed a tumor composed of anastomosing vascular channels in an infiltrating growth pattern. The vascular channels are lined by atypical endothelial cells with enlarged hyperchromatic nucle (black arrow).

**Figure 4:**
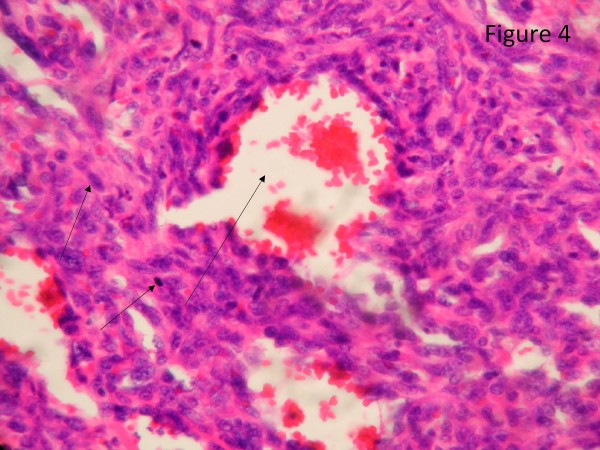
**The section of resected liver showed spindled tumor cells forming hemorrhagic nests and sheets**. The section of resected liver showed spindled tumor cells forming hemorrhagic nests and sheets. Rudimentary vascular channels were noted within the tumor cell nest. The spindled tumor cells showed high grade nuclear atypia with brisk mitotic activity(black arrows).

**Figure 5:**
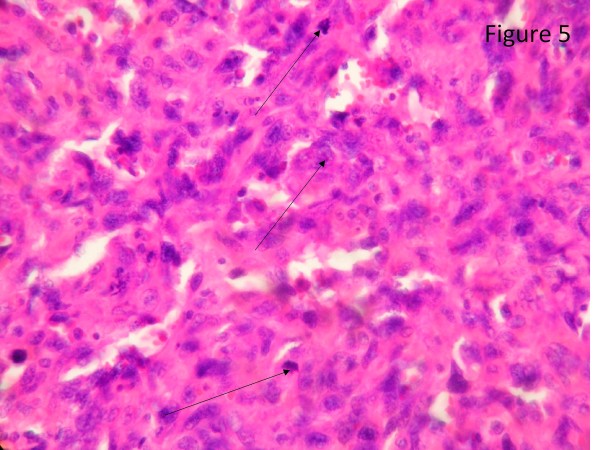
**Epithelioid areas**. Epithelioid areas made up of large rounded tumor cells with high grade nuclear atypia and brisk mitotic activity were also seen(black arrows).

**Figure 6:**
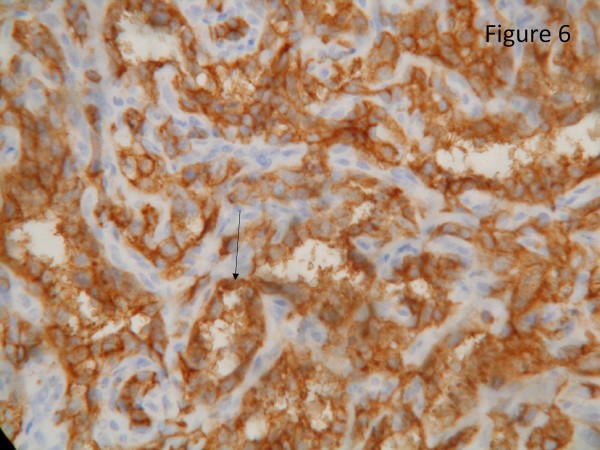
**Immunohistochemical study**. Immunohistochemical study showed that the tumor cells expressed vascular antigen CD31 strongly(black arrow, score 3+, clone 1A10, Novocastra).

**Figure 7:**
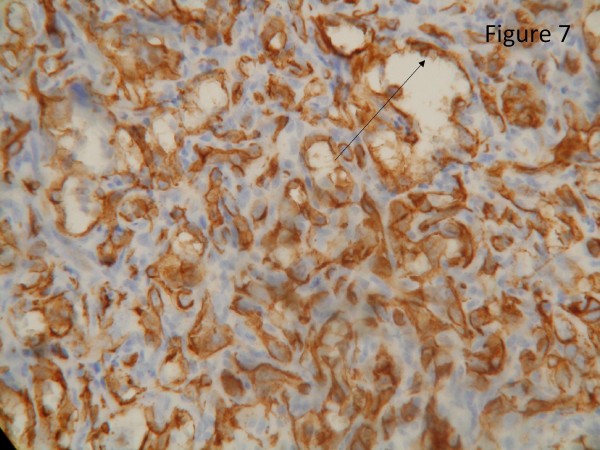
**Immunohistochemical study**. Immunohistochemical study revealed that the tumor cells was strongly positive for vascular antigen CD34 (black arrow, score 3+, clone QBEnd-10, Dako).

**Figure 8:**
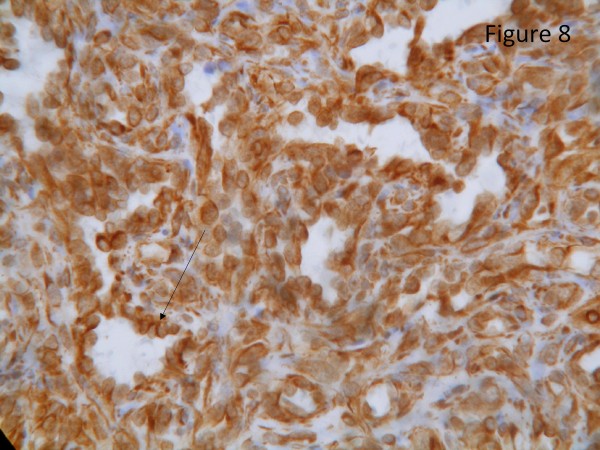
**Immunohistochemical study**. Immunohistochemical study demonstrated that the tumor cells strongly expressed the mesenchymal marker, Vimentin (black arrow, score 3+, clone V9, Novocastra).

**Figure 9:**
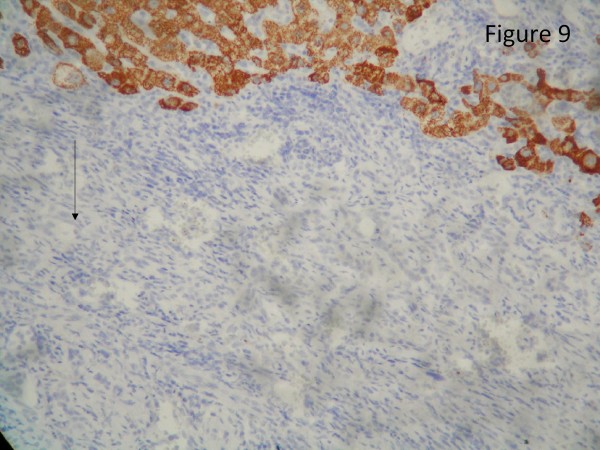
**Immunohistochemical study**. Immunohistochemical study revealed negative staining of hepatocyte paraffin antibody (HAS, clone OCH1E5, CellMarque) over tumor cells (black arrow).

**Figure 10:**
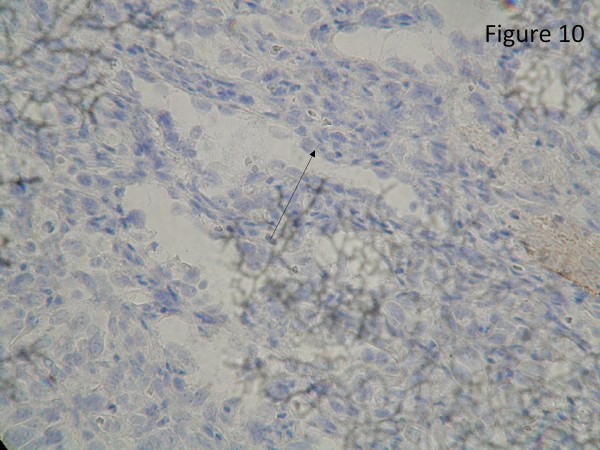
**Immunohistochemical study**. Immunohistochemical study showed negative staining of Glypican-3 (GPC-3, clone 1G12, Bio SB) over tumor cells (black arrow).

The resected liver contained a tumor composed of anastomosing vascular channels in an infiltrating growth pattern. The vascular channels were lined by atypical endothelial cells with enlarged hyperchromatic nuclei (Figure 3). The other sections of resected liver showed spindled or epithelioid tumor cells forming hemorrhagic nests and sheets with rudimentary vascular channels. The tumor cells possessed high grade nuclear atypia and brisk mitotic activity(Figure 4). Epithelioid areas made up of large rounded tumor cells with high grade nuclear atypia and brisk mitotic activity were also seen (Figure 5). Immunohistochemically, the tumor cells expressed usual vascular antigens CD31 and CD34 as well as mesenchymal marker, Vimentin (Figure 6, 7, 8) [9,10]. The negative staining of HAS and GPC-3 excludes the possibility of hepatocytic neoplasm(Figure 9, 10) [11,12]. Subsequently, a final diagnosis of liver angiosarcoma was confirmed.

After operation, this patient recovered very well and was discharged two weeks later. Adjuvant chemotherapy and radiation were not given after taking the effectiveness of them and her old age into consideration. No local recurrence or distant metastasis has been found at the time of writing, 4 months after operation. This patient is now still with a good performance status.

## Discussion

Angiosarcoma, a subtype of soft tissue sarcoma, is an aggressive malignant disease deriving from endothelium, lymphatics, or blood vessels. Angiosarcoma occurs most commonly in head and neck, followed by breast. Liver angiosarcoma is ranked as fifth in the list of most common seen sites of angiosarcoma [9] and is very rare.

Liver angiosarcoma occurs in the elder mainly [1]. Although the symptoms are usually nonspecific, abdominal distension and discomfort, weight loss, and fatigue are commonly found [13,14]. Others like jaundice, ascites, or hepatomegaly are usually associated with advanced liver angiosarcoma [13,15]. The abnormal liver functions found in advanced liver angiosarcoma are attributable to the replacement of normal liver parenchema by tumor [16].

Due to the hypervascular characteristic of liver angiosarcoma, it is difficult to differentiate liver angiosarcoma from other vascular tumors in liver, such as hepatoma or adenoma, radiologically. As shown in the present case, CT and angiogram all indicated a hypervascular mass, which was in line with the image findings of hepatoma. After resection, pathologically, the resected liver showed a tumor composed of anastomosing vascular channels infiltrating surrounding hepatic tissue in a destructive fashion. The channels were lined by atypical endothelial cells with enlarged hyperchromatic nuclei. Immunohistochemically, the tumor was positive for CD31, CD34, and Vimentin, a mesenchymal marker, and negative for HAS and GPC-3, which excluded the possibility of hepatocyte origin. Taken together, the liver angiosarcoma was confirmed as the final diagnosis [9-12].

The survival of patients with liver angiosarcoma is very poor with media survival of 6 months without treatment. Even after treatment, only 3% of patients were reported to live longer than 2 years [3]. The reasons contributing to this are complicated. First, due to its nonspecific symptoms, the diagnosis is usually too late, which excludes the chance of complete resection of tumor in most cases. Second, liver angiosarcoma usually presents with early metastases to other organs, such as lung, spleen, or bone, which further worsens its prognosis. At present, the standard treatment for liver angiosarcoma remains surgical resection. Others such as chemotherapy or radiotherapy fails to reach a conclusive benefit on survival of liver angiosarcoma [5-7], although chemotherapy is the critical part of treatments of soft tissue sarcoma and radiotherapy plays a palliative role in liver metastasis [17]; Liver transplantation is also not deemed feasible to treat liver angiosarcoma thanks to its high recurrent rate and still poor survival after transplantation [8].

Liver angiosarcoma also easily induces severe intraabdominal hemorrhage due to spontaneous rupture, leading to subsequent tumor cell spillage and peritoneal tumor seeding, which further complicates the prognosis [18,19]. Once liver angiosarcoma-induced internal bleeding happens, TAE is usually the first choice to stop the bleeding and to get the patients stable [18,19]. Otherwise, in relatively stable patients, conservative treatment may also work in this situation [2]. However, due to the rare incidence of liver angiosarcoma, how to treat acute bleeding of liver angiosarcoma has not yet reached a definite conclusion at present. As shown in the present case, spontaneous liver angiosarcoma rupture made her present with a shock status at first. After embolizaton and resuscitation, her condition got stabilized and she received partial hepatectomy two weeks later. For this patient, the poor prognosis is expected according to previous reports. However, given that this patient is already 83 years old and no effective strategies against liver angiosarcoma are available so far, no more adjuvant therapy has been given to this patient. No recurrence or metastasis has been identified at the time of writing and she is still with a good performance status so far, 4 months after operation.

## Conclusion

Seeking a new strategy in addition to surgical resection to treat liver angiosarcoma should be prioritized due to its high recurrence rate and very dismal prognosis. Since traditional chemotherapy and radiotherapy could not benefit this kind of patients, combined with the rapid progress of current molecular biology, target therapy may be the further direction against liver angiosarcoma

## Consent statement

This study has been approved by Chang Gung memorial hospital IRB board. The approved IRB number is 100-3732B. A copy of the approval of IRB is available for review by the Editor-in-Chief of this journal.

## Competing interests

The authors declare that they have no competing interests.

## Authors' contributions

CY wrote the manuscript, Cheng-cheng carried out the pathological examination, Chun-nan Yeh helped wrote the manuscript, Huang-yang helped wrote the manuscript, Jui- Teng participated in data collection, Chan-Siu carried out the image reading, Chih-Lang helped data collection, Cho-li participated in pathological examiantion, Wen-yen participated in data collection, Kun-Chun finalized the manuscript. All authors read and approved the final manuscript.
